# Age influences structural brain restoration during weight gain therapy in anorexia nervosa

**DOI:** 10.1038/s41398-020-0809-7

**Published:** 2020-05-04

**Authors:** Lisa-Katrin Kaufmann, Jürgen Hänggi, Lutz Jäncke, Volker Baur, Marco Piccirelli, Spyros Kollias, Ulrich Schnyder, Chantal Martin-Soelch, Gabriella Milos

**Affiliations:** 1 Department of Consultation-Liaison Psychiatry and Psychosomatics, University Hospital Zurich, University of Zurich, Zurich, Switzerland; 2 Division of Neuropsychology, Department of Psychology, University of Zurich, Zurich, Switzerland; 3 Unit of Clinical and Health Psychology, Department of Psychology, University of Fribourg, Fribourg, Switzerland; 4 International Normal Aging and Plasticity Imaging Center (INAPIC), University of Zurich, Zurich, Switzerland; 5 University Research Priority Program (URPP) “Dynamic of Healthy Aging”, University of Zurich, Zurich, Switzerland; 6 Department of Neuroradiology, University Hospital Zurich, Zurich, Switzerland; 7 University of Zurich, Zurich, Switzerland

**Keywords:** Psychiatric disorders, Neuroscience

## Abstract

Neuroimaging studies on anorexia nervosa (AN) have consistently reported globally reduced gray matter in patients with acute AN. While first studies on adolescent AN patients provide evidence for the reversibility of these impairments after weight gain, longitudinal studies with detailed regional analysis for adult AN patients are lacking and factors associated with brain restitution are poorly understood. We investigated structural changes in anorexia nervosa using T1-weighted magnetic resonance images with surface-based morphometry. The sample consisted of 26 adult women with severe AN and 30 healthy controls. The longitudinal design comprised three time points, capturing the course of weight-restoration therapy in AN patients at distinct stages of weight gain (BMI ≤ 15.5 kg/m^2^; 15.5 < BMI < 17.5 kg/m^2^; BMI ≥ 17.5 kg/m^2^). Compared to controls, AN patients showed globally decreased cortical thickness and subcortical volumes at baseline. Linear mixed effect models revealed the reversibility of these alterations, with brain restoration being most pronounced during the first half of treatment. The restoration of cortical thickness of AN patients negatively correlated with age, but not duration of illness. After weight restoration, residual group differences of cortical thickness remained in the superior frontal cortex. These findings indicate that structural brain alterations of adult patients with severe AN recuperate independently of the duration of illness during weight-restoration therapy. The temporal pattern of brain restoration suggests a decrease in restoration rate over the course of treatment, with patients’ age as a strong predictor of brain restitution, possibly reflecting decreases of brain plasticity as patients grow older.

## Introduction

Anorexia nervosa (AN) is a severe and enduring psychiatric disorder, characterized by significantly reduced body weight^1^ and associated with reduced brain matter^2,3^. In order to better understand the hitherto unclear psychopathology of the illness, these brain alterations are being examined more closely using magnetic resonance imaging (MRI).

Recent studies on adult AN patients have largely established reduced gray matter volume in a globally distributed set of brain regions, affecting almost the whole cortex^3–5^. Reports on reduced white matter volumes have been less consistent, with some studies reporting decreased volume^6,7^, while others did not find significant changes^8,9^. These reductions are generally interpreted as consequences of patients’ malnutrition and are mostly absent in long-term recovered AN patients^10–15^. However, reports on persevering gray matter decreases in regions such as the precuneus^11^, the anterior cingulate cortex, and the supplementary motor area^16^, as well as persisting volume increases in the insula, the orbitofrontal cortex^17,18^, and the post-central gyrus^19^ paint a more complex picture. Longitudinal study designs are thus required to address the question of brain regeneration in women with AN. In adolescent AN patients and a mixed-sample of adolescents and young adults, three longitudinal studies report complete normalization of gray matter loss after a brief period of weight restoration^20–22^. For adult AN patients, only global tissue volumes have been investigated^23,24^, but longitudinal studies with detailed regional analyses are lacking.

In this longitudinal study, we aimed to identify and monitor disease-specific structural brain alterations in patients with severe AN using three time points: (TP1) at the beginning of inpatient treatment in a stage of severe underweight, (TP2) during inpatient treatment after initial weight gain, and (TP3) at discharge with a body mass index ≥ 17.5 (BMI; kg/m^2^). Based on previous results^3,4^, we expected decreases in cortical and subcortical gray matter of AN patients in the acute stage compared to healthy controls (HC). Over the course of weight-restoration treatment, we expected a regeneration of these gray matter compared to HC. Additionally, we explored whether restoration of brain structure in AN patients was associated with a shorter duration of illness, with improvements in BMI, eating disorder-related cognitions, or with a younger age at the time of treatment.

## Materials and methods

### Participants

We recruited 26 women diagnosed with severe AN (22 restrictive, 4 binge/purge; 18−32 years) and 30 HC (BMI 18.5−23.0 kg/m^2^; 18−30 years). Inclusion criteria for the AN group comprised severe underweight (BMI ≤ 15.5 kg/m^2^) to allow for a homogeneous weight development and to capture brain changes at distinct phases of weight gain (see “Study design”). Both groups were assessed with the Structured Clinical Interview for DSM-IV-TR Axis I Disorders^25^ and matched for sex, age, handedness, intelligence and years of education. All women were strongly right-handed, except for one left-handed woman in each group^26^. Fifteen patients were given comorbid diagnoses: major depression (12), major depression and social anxiety disorder (2), and obsessive-compulsive disorder (1). Twelve patients were receiving medication when they entered the study; these patients were instructed to continue taking them as prescribed (see Table S1 for further details). Exclusion criteria comprised current or past neurological disorders, substance abuse or addiction, contraindications to MRI, and for HC a history of eating disorders or any mental illness, first- or second-degree relatives with a lifetime diagnosis of an eating disorder, and the use of any medication, including hormonal contraceptives.

Data of two patients were not available at the first time point due to technical difficulties. This sample partially overlaps with the sample of a recent publication of our group, focusing on white matter alterations in the fornix^27^. The study was approved by the local ethics committee and the study protocol complied with the Declaration of Helsinki. All participants gave their written informed consent prior to study enrollment.

### Study design

Patients with AN participated in an eating disorder-specific inpatient therapy program with a target BMI ≥ 18.5 kg/m^2^. Measurements of the AN group were taken at three time points: at the beginning (TP1; BMI ≤ 15.5 kg/m^2^), in the middle (TP2: 15.5 < BMI < 17.5 kg/m^2^), and towards the end of treatment with a BMI of ≥17.5 kg/m^2^ (TP3; see Supplement for further details). The HC group participated in the same procedure at two time points (TP1 and TP3). The time interval between TP1 and TP3 for both groups was 4-6 months after TP1 (see also Supplement). The patients’ TP1 was scheduled after a medical stabilization period of at least 2 weeks with a fixed meal plan and extensive somatic checks, to exclude biases of under- or hyperhydration due to the condition of acute starvation^28,29^.

### Behavioral and psychometric assessment

Validated German versions of the following psychometric tests were used in this study: The Viennese Matrices Test (WMT) for non-verbal intelligence^30^ and the Vocabulary Test (WST) for verbal intelligence^31^, both presented as computer-based versions^32^. Eating disorder cognitions were assessed with the Eating Disorder Examination Questionnaire (EDE-Q)^33^, depression severity was captured with the Beck Depression Inventory (BDI)^34^.

### MRI data acquisition

Brain images were acquired with a 3.0 Tesla whole-body MRI system (Ingenia, Philips, Best, The Netherlands), equipped with a 32-channel head coil. Whole-brain 3D T1-weighted structural images were acquired using a 3D Turbo-Field-Echo sequence (for details, see Supplement). All images were checked for relevant clinical pathology or anomalies by a trained neuroradiologist.

### MRI data preprocessing

Structural T1-weighted images were preprocessed with the FreeSurfer software suite version 6.0.0 (http://surfer.nmr.mgh.harvard.edu/). This automated analysis procedure has been widely used and its preprocessing steps have been described in detail elsewhere^35^. In brief, T1-weighted images in stereotactic space are segmented into different tissue types, considering a priori anatomical information and each voxel’s intensity value. To estimate cortical thickness and subcortical volumes, images were preprocessed with FreeSurfer’s longitudinal stream^36^. For each subject an unbiased median template image was created^37^ using robust, inverse consistent registration between each time point of a subject^38^. Data for each subject were then resampled to the respective template and preprocessed by (1) registration to Talairach space, (2) construction of brain mask and skull stripping, (3) normalization and registration to the probabilistic atlas^39,40^, (4) segmentation of subcortical regions, (5) creation of spherical surface maps, (6) registration to cortical atlas, and (7) parcellation, initialized with common information from the within-subject template to improve statistical power and reliability^36^. For each time point, cortical thickness maps for both hemispheres were then extracted and smoothed with a 15-mm full-width at half-maximum Gaussian kernel. Additionally, global brain measures and subcortical structures (averaged over both hemispheres) provided by the FreeSurfer stream were investigated. To assure data quality, all preprocessed images were visually inspected and checked with FreeSurfer’s quality assessment tool.

### MRI data analysis

The longitudinal analyses of the cortical thickness were performed using linear mixed effects (LME) models, more specifically with the spatiotemporal extension for mass-univariate data as distributed with FreeSurfer^41,42^. LME models offer a powerful and versatile approach for the analysis of longitudinal data, with the advantage of differentiating between-subject and within-subject sources of variance and handling unequal numbers of time points^41^. Visual inspection of mean trajectories revealed a linear trend over time; thus, the spatiotemporal model was fitted with the intercept as random effect. For each tested contrast (main effect group, main effect time, interaction effect group × time), significance maps were created, and false discovery rate corrected within FreeSurfer (across both hemispheres) at a significance level of *p* < 0.05, using an adaptive linear two-stage procedure^43^ to control for multiple comparisons. Time was measured in days from TP1. The longitudinal analyses for subcortical volumes were conducted using univariate LME models implemented in FreeSurfer, with intracranial volume as covariate and the intercept as random effect. To assess the magnitude and direction of differences, these comparisons were followed up by paired *t-*tests within groups. Reported *p-*values are adjusted for multiple comparisons using the Holm−Bonferroni procedure^44^.

Cross-sectional comparisons of cortical thickness between groups at TP1 and TP3 were calculated using vertex-wise general linear models with group as independent and cortical thickness as dependent variable within FreeSurfer, using Monte-Carlo simulation (5000 permutations) to correct for multiple comparisons. To perform cross-sectional comparisons of volumetric measures, values for global cortical and subcortical volumes were extracted from FreeSurfer. Subcortical volumes were corrected for intracranial volume ((volume/intracranial volume) × 1000). To check for normality of the data distribution, the Shapiro Wilk test was used. Group comparisons were performed using *t* tests for independent samples, adjusted for multiple comparisons (separately for regional and global measures) using the Holm-Bonferroni procedure^44^. All *t-*tests were performed using R, version 3.5.0 ^45^.

### Additional statistical analyses

Within- and between-group comparisons of demographic and psychometric measures were performed using two-tailed *t-*tests for paired or independent samples, respectively. To check for normality of the data distribution, the Shapiro-Wilk test was used. Standardized mean differences are reported as Hedges’ *g*, with the pooled standard deviation as standardizer^46^ (see Supplement for details). Associations between changes in gray matter (cortical thickness per hemisphere, cortical thickness within the clusters of reduced cortical thickness identified at TP1, subcortical volumes) and clinical parameters (BMI, BMI increase per week, duration of illness, eating disorder-related cognitions, and depression severity) as well as age (at the beginning of treatment) were calculated as Pearson correlations. These tests were performed using R, version 3.5.0 ^45^ and subsequently adjusted for multiple comparisons according to Holm-Bonferroni^44^.

## Results

### Demographic and psychometric measures

Group comparisons of demographic data at baseline (TP1) revealed no significant differences between AN patients and HC with respect to age, intelligence and education (Table 1). As expected, AN patients had significantly lower BMIs, higher eating disorder-related cognition (EDE-Q) and depression (BDI) scores than HC at TP1. Over the course of therapy, patients’ BMI increased significantly compared to baseline (TP1 to TP2, 16% BMI increase; TP2 to TP3, 13% BMI increase). Similarly, psychometric scores of the AN group improved and dropped below the clinical cut-offs at the end of treatment, but remained significantly elevated compared to HC (Table 1).

**Table 1:** Group characteristics.

	Group and time point										AN1 vs. HC1	AN3 vs. HC3	AN1 vs. AN3
	AN1 (*n* = 24)		AN2 (*n* = 26)		AN3 (*n* = 26)		HC1 (*n* = 30)		HC3 (*n* = 30)				
	*M*	*SD*	*M*	*SD*	*M*	*SD*	*M*	*SD*	*M*	*SD*	*p*	*p*	*p*
Age	22.25	4.08	–	–	–	–	24.15	3.42	–	–	0.075	–	–
Age of illness onset	16.70	3.08	–	–	–	–	–	–	–	–	–	–	–
Duration of illness	5.59	4.30	–	–	–	–	–	–	–	–	–	–	–
Education	13.30^a^	2.53	–	–	–	–	14.69^b^	4.68	–	–	0.180	–	–
BMI (kg/m^2^)	14.25	1.08	16.55	0.68	18.41^c^	0.45	20.81	1.77	20.38	1.56	<0.001	<0.001	<0.001
BMI increase per week	–	–	0.24	0.07	0.10	0.04	–	–	−0.01	0.04	–	<0.001	–
Time after TP1 (weeks)	–	–	9.16	4.16	22.04	16.26	–	–	25.91	8.66	–	0.283	–
EDE-Q total	3.19	1.26	1.98	1.11	1.57^a^	1.01	0.64^b^	0.58	0.55^d^	0.57	<0.001	<0.001	<0.001
EDE-Q eating concern	3.16	1.33	1.84	1.15	1.41^a^	1.11	0.43^b^	0.56	0.41^d^	0.56	<0.001	<0.001	<0.001
EDE-Q restraint	3.29	1.82	1.33	1.24	1.10^a^	0.81	0.48^b^	0.60	0.28^d^	0.39	<0.001	<0.001	<0.001
EDE-Q shape concern	3.47	1.22	2.85	1.57	2.27^a^	1.50	0.91^b^	0.88	0.84^d^	0.76	<0.001	<0.001	<0.001
EDE-Q weight concern	2.84	1.21	1.90	1.10	1.50^a^	1.28	0.75^b^	0.75	0.67^d^	0.80	<0.001	0.012	<0.001
BDI	22.83	9.93	17.77	9.40	10.74^a^	7.93	3.48^b^	3.82	2.37^d^	2.51	<0.001	<0.001	<0.001
WMT	121.13	17.64	–	–	–	–	127.83	15.81	–	–	0.153	–	–
WST	105.26^a^	8.24	–	–	–	–	105.70	10.43	–	–	0.865	–	–

Age, age of illness onset, duration of illness, and education in years.

*BMI* body mass index, *BDI* Becks Depression Inventory total score (cut-off for clinical range ≤ 11^66^), *EDE-Q* Eating Disorder Examination Questionnaire score (cut-off for clinical range ≥ 2.09^67^), *WMT* Viennese Matrices Test, *WST* Multiple Choice Vocabulary Test.

^a^Data available only for AN = 23

^b^HC = 29 subjects

^c^BMI ≥ 18.5: *n* = 15.

^d^HC = 27 subjects.

### Brain alterations during severe underweight (TP1)

Global brain volumes at TP1 showed reduced cortical gray matter and heightened CSF in AN patients compared to HC, and a trend for reduced global subcortical gray matter (Table 2). No differences were observed with respect to cerebral white matter. Furthermore, intracranial volumes did not differ between groups, ruling out overall smaller skulls in AN patients as explanation for the observed differences.

**Table 2:** Comparison of cortical and subcortical brain volumes per group and time point (cm^3^).

	Group and time point										AN1 vs. AN2	AN2 vs. AN3	HC1 vs. HC3	AN1 vs. HC1	AN3 vs. HC3
	AN1 (*n* = 24)		AN2 (*n* = 26)		AN3 (*n* = 26)		HC1 (*n* = 30)		HC3 (*n* = 30)						
	*M*	*SD*	*M*	*SD*	*M*	*SD*	*M*	*SD*	*M*	*SD*	*g*	*g*	*g*	*g*	*g*
Gray matter, cortex	448.57	27.22	468.49	32.88	476.62	34.85	473.37	38.23	471.80	35.23	0.66***	0.24***	−0.04	0.73*	−0.14
Gray matter, subc.	57.38	3.79	58.46	3.80	58.90	3.98	59.99	4.03	59.85	3.98	0.28***	0.11**	−0.03	0.66°	0.24
White matter	438.10	35.55	440.29	36.59	442.73	37.17	450.82	51.94	447.36	50.06	0.06	0.07	−0.07	0.28	0.10
Cortico-spinal fluid	23.97	10.01	21.58	8.81	20.77	8.89	15.61	4.08	15.64	4.11	−0.25***	−0.09**	0.01	−1.14**	−0.76°
Intracranial volume	1549.12	123.27	1557.25	137.46	1557.25	137.46	1523.39	159.25	1523.39	159.25	0.06	0.00	0.00	−0.18	−0.23
Accumbens nucleus	0.40	0.08	0.40	0.08	0.41	0.08	0.45	0.09	0.45	0.08	−0.08	0.06	−0.02	0.56	0.62
Amygdala	1.57	0.15	1.61	0.14	1.61	0.15	1.63	0.13	1.62	0.13	0.17**	0.02	−0.03	0.49	0.28
Caudate nucleus	3.59	0.42	3.73	0.43	3.75	0.46	3.80	0.42	3.76	0.41	0.26*	0.04	−0.08	0.57	0.23
Hippocampus	4.12	0.38	4.21	0.36	4.26	0.37	4.40	0.34	4.42	0.37	0.19***	0.13**	0.03	0.92*	0.66
Pallidum	1.97	0.14	1.97	0.14	1.97	0.12	2.02	0.23	2.04	0.23	−0.07	−0.01	0.11	0.38	0.58
Putamen	4.93	0.49	4.99	0.46	4.98	0.50	5.10	0.53	5.07	0.53	0.06	−0.01	−0.05	0.46	0.38
Thalamus	7.36	0.45	7.59	0.53	7.70	0.55	7.81	0.64	7.79	0.62	0.24***	0.14***	−0.04	0.83*	0.34

Mean values represent absolute values. Individual subcortical volumes were averaged across hemispheres and comparisons were performed with mean values corrected for total intracranial volume.

*g* Hedges’ *g*, *subc.* subcortical.

°*p* < 0.10, *p < 0.05, **p < 0.01, ***p < 0.001 adjusted for multiple comparisons according to Holm−Bonferroni.

**Fig. 1:**
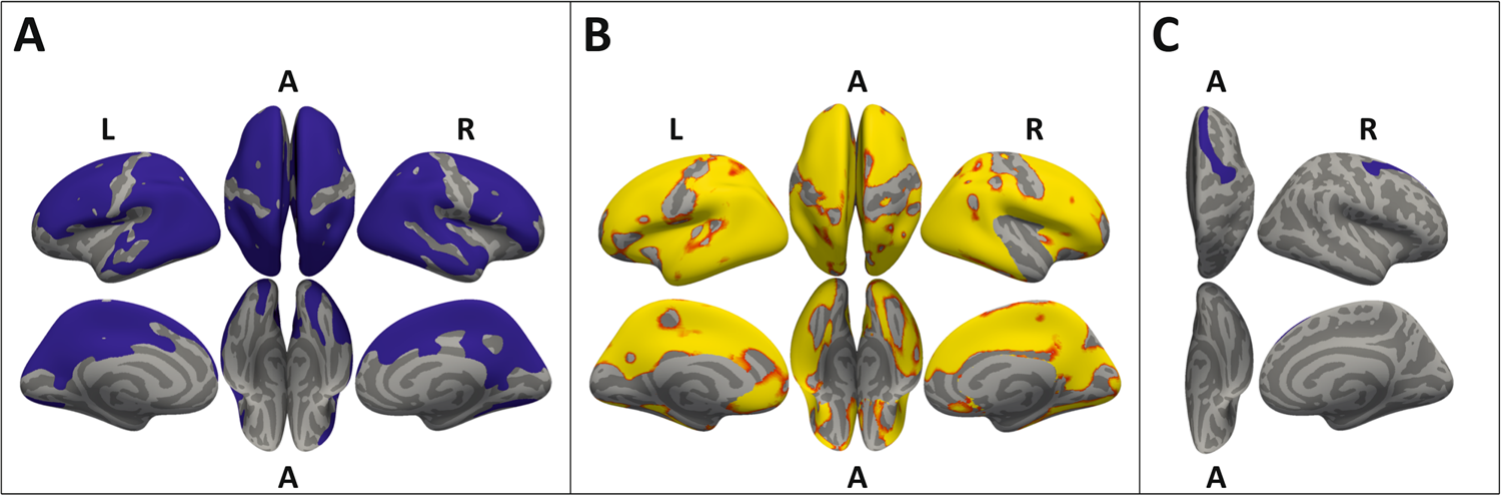
Group comparisons of cortical thickness projected onto inflated brain surfaces (gray). **a** Reduced cortical thickness of AN patients compared to HC at the beginning of treatment (TP1). Significant clusters (blue, *p* < 0.05), corrected for multiple comparisons using Monte-Carlo simulations (5000 permutations). **b** Increase of cortical thickness during weight restoration in AN patients. Significant interaction of group × time (red, *p* < 0.05; yellow, *p* < 0.01), controlled for multiple comparisons with FDR correction. **c** Residual cortical thinning in AN patients compared to HC at the end of treatment (TP3). A significant cluster (blue, *p* < 0.05) remained in the superior frontal region (AN: mean = 2.71 mm ± 0.15, HC: mean = 2.86 mm ± 0.19), after correcting for multiple comparisons using Monte-Carlo simulation (5000 permutations). A anterior, L left, R right.

Vertex-wise group comparisons of cortical thickness at TP1 demonstrated that global volumetric gray matter differences reported above were likely driven by differences in cortical thickness. Compared to HC, AN patients showed considerably decreased cortical thickness in clusters covering large parts of both hemispheres (Fig. 1a), sparing only parts of the medial prefrontal, sensorimotor, and insular cortex, as well as basal parts of the temporal lobe and the calcarine cortex. Separate analyses of the subcortical structures yielded reduced volumes in patients with AN compared to HC at TP1, with pronounced differences in the hippocampus and the thalamus (Table 2).

### Recovery processes with weight restoration

**Fig. 2:**
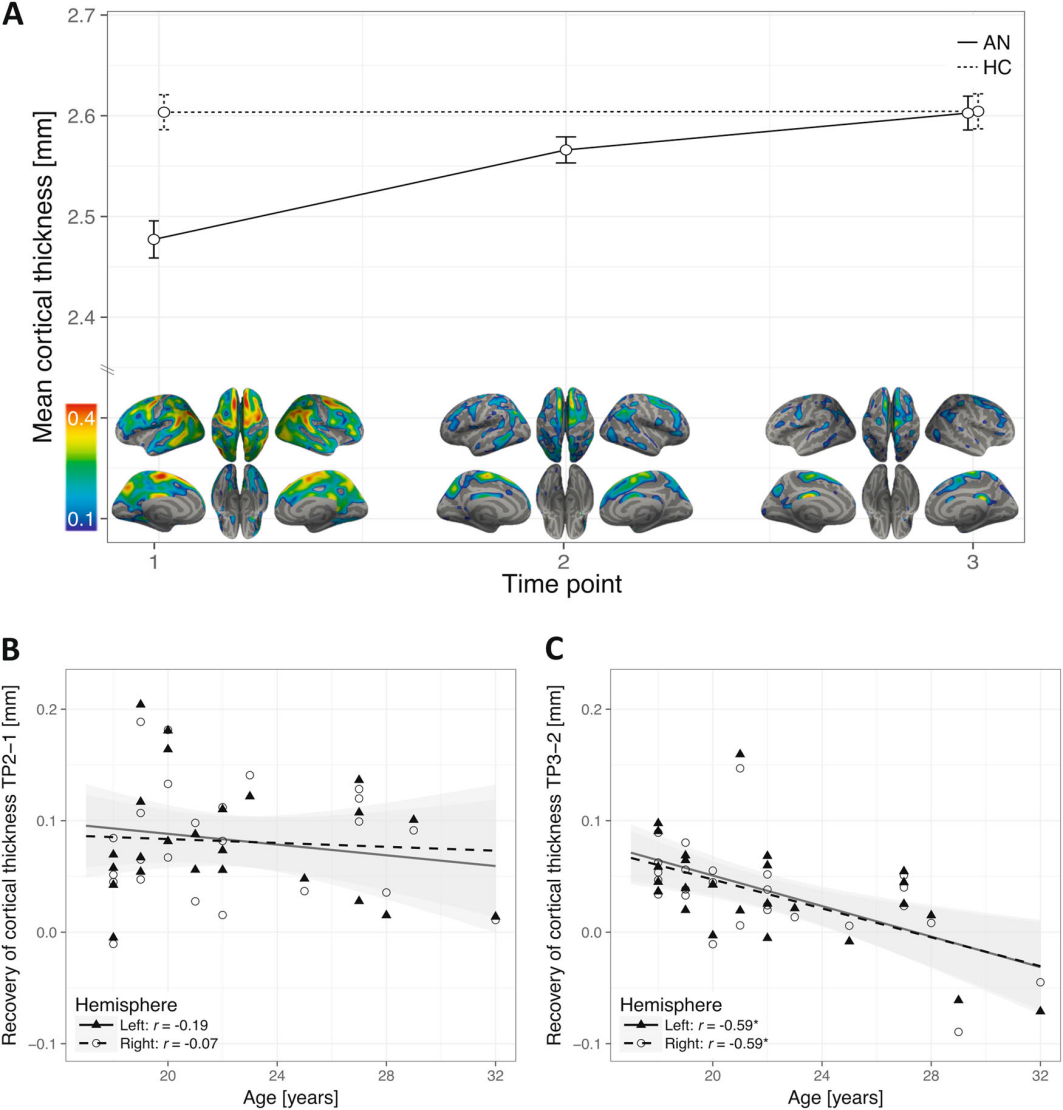
**Recovery of cortical thickness and correlation with patients**’ **age. a, top**: Global cortical thickness (mm) per group, left hemisphere displayed (similar trajectory for right hemisphere, not displayed). Significant increase within AN group during first (TP2-TP1: mean increase left = 0.08 mm ± 0.05, t[23] = 7.7, *p* < 0.001; mean increase right = 0.08 mm ± 0.05, t[23] = 7.7, *p* < 0.001) and second (TP3-TP2: mean increase left = 0.04 mm ± 0.05, t[25] = 4.0, *p* < 0.001; mean increase right = 0.03 mm ± 0.04, t[25] = 3.9, *p* < 0.001) treatment phase. Larger increase during first phase than during second phase (left: mean difference of increase = 0.045 mm ± 0.078, t[23] = 2.81, *p* < 0.001; right: mean difference of increase = 0.048 mm ± 0.077, t[23] = 3.06, *p* < 0.001). Error bars represent 95% within-subject confidence intervals^68^. **a**, **bottom**: Local differences in cortical thickness between groups (HC-AN) per time point, projected onto inflated brain surfaces (gray). HC values were averaged over time before differences between groups were calculated. **b** No significant correlation between age and changes in cortical thickness of AN patients between TP1 and TP2. **c** Significant correlations between age and recovery of cortical thickness in AN patients between TP2 and TP3; left hemisphere *R*^2^ = 0.35, right hemisphere *R*^2^ = 0.35. *p < 0.05, Holm−Bonferroni corrected.

LME analyses of cortical thickness yielded a significant interaction of group × time in each hemisphere, revealing cortical restoration in AN patients over the course of treatment (Fig. 1b). Within-group analysis demonstrated significant global restoration of cortical thickness in both hemispheres during the first (mean increase = 0.08 mm) and second treatment phase (mean increase = 0.04 mm), with a notably faster restoration during the first phase (Fig. 2a). Despite the significant global restoration of cortical thickness (Fig. 1b), whole-brain comparisons at the end of treatment (TP3) yielded a significant cluster of residual cortical thinning in AN patients compared to HC in the right superior frontal cortex (Fig. 1c).

LME analyses of subcortical volumes yielded significant interactions of group and time for the following structures: amygdala, caudate nucleus, hippocampus, and thalamus (*p*s ≤ 0.002, corrected; Supplementary Table S2). Within-group comparisons of the subcortical structures demonstrated statistically significant volume increases in AN patients of the amygdala, caudate nucleus, hippocampus, and thalamus during the first phase of treatment. In the second phase of treatment, only volumes of the hippocampus and the thalamus further increased (Table 2). Volumes of the nucleus accumbens, pallidum, and putamen did not change significantly within the AN group, as well as in comparison with HC (Table 2, Supplementary Tables S2, S3). Group comparisons at TP3 yielded no longer significant differences in subcortical volumes, albeit slight remaining decreases (Table 2). There was no evidence that recovery processes were driven by AN subtype or patients’ medication status (Supplementary Fig. S1, S2).

### Correlations with clinical and demographic parameters

During the early phase of treatment (TP1−TP2), the recovery of subcortical structures was not associated with changes in clinical parameters, except for a positive association between the regeneration of the caudate nucleus and BMI increase (*r* = 0.68, *p* < 0.01, corrected; Supplementary Table S4). Similarly, BMI increase correlated with changes of cortical thickness of the right hemisphere on a trend level (*r* = 0.59, *p* = 0.06, corrected; Table S5). Considering the homogeneity of BMI changes within the AN patient group, these associations underline the strong influence of body weight on structural brain measures.

During the later phase of treatment (TP2−TP3), the restoration of cortical thickness in both hemispheres was strongly correlated with patients’ age, suggesting that patients’ cortical thickness regenerated to a larger degree if patients were younger (Fig. 2b, c). Of note, this relationship persisted even after controlling for BMI at TP3 (left hemisphere, *r* = −0.60, *p* = 0.001; right hemisphere, *r* = −0.61, *p* < 0.001) or the change of BMI between TP2 and TP3 (left hemisphere, *r* = −0.60, *p* = 0.001; right hemisphere, *r* = −0.60, *p* = 0.001). Furthermore, the reduction of cortical thickness at TP1 was independent of patients’ age (Supplementary Fig. S3), indicating that younger patients’ cortices were not thinner to start with. Other parameters were not associated with the increase of cortical thickness or subcortical volumes during this later phase of treatment.

## Discussion

This longitudinal study investigated temporal patterns of cortical and subcortical regeneration in women with severe AN, assessing brain restoration at three distinct stages of weight gain. In the stage of severe underweight, our analyses revealed reduced cortical thickness as well as diminished subcortical volumes, which largely regenerated during a substantial weight gain up to a BMI of ≥17.5 kg/m^2^. Specifically, patients’ mean cortical thickness during the early phase of treatment increased markedly by 0.08 mm (TP1 to TP2, 16% BMI increase compared to baseline). During the later phase of treatment, further restitution of mean cortical thickness by 0.04 mm was observed (TP2 to TP3, 13% BMI increase compared to baseline). However, significant residual differences remained in a right superior frontal cluster. Closer examination of associated factors revealed a strong influence of age and BMI on cortical gray matter regeneration, suggesting the cortex of younger patients recovered to a larger extent during weight normalization. Apart from highlighting the impact of body weight on the structural measures of the brain, this may indicate a decrease of brain plasticity as patients grow older^47^.

The reversibility of decreases in cortical thickness and subcortical volumes is in overall agreement with findings of longitudinal studies in adolescent AN patients after a brief period of weight restoration^20,21^. In adolescents, BMI increases of at least 10% were accompanied by an average restitution of cortical thickness of 0.16 mm after a treatment duration of 12 weeks^20^. In comparison, the increases of cortical thickness in our sample added up to only 0.12 mm after a total treatment duration of 22 weeks. Recovery processes in adolescent AN patients might thus happen at a faster rate compared to adult patients. An alternative explanation for the differences in restoration rate might be the longer duration of illness and higher disease severity (indexed by the BMI) of patients in the current sample. However, this is not supported by the lack of correlation between illness duration and the regeneration of cortical thickness. Additionally, our finding of a persisting influence of age, after controlling for BMI and change in BMI, speaks against such an interpretation. An initially greater reduction of cortical thickness in younger patients may be another reason for the differing velocities of regeneration. However, we did not find evidence for an association between age and cortical thickness during the stage of acute starvation in the present sample. Lastly, the observed restoration rate might be influenced by the medical stabilization period before TP1 we used to mitigate inflated effects, which was not employed in the studies with adolescents. However, hydration status in adolescents was reported to be within the normal range^20^. Moreover, the residual cortical thinning at TP3 in our sample, compared to fully normalized gray matter values in adolescents^20,21^, indicates that the time-lag between admission and TP1 does not suffice to explain the lower recovery rate in adults.

Cross-sectional comparisons at TP3 demonstrated remaining decreases of cortical thickness in the superior frontal region. The residual gray matter reduction is in line with another study investigating weight restored adult AN patients, which reported persisting global reductions after reaching 90% of the ideal body weight^23^. However, the authors of the study did not separately analyze the influence of patients’ age. In long-term recovered adult AN patients, age has not been found to be associated with gray matter measures^10,48^. Together with the considerable influence of age on brain restoration only in the latter part of treatment, this could indicate that brain regeneration in relatively older patients is slower, rather than incomplete, compared to younger patients.

Similar to the trajectory of cortical regeneration, the restoration of subcortical volumes showed the largest effects during the early phase of treatment with substantial increases of the amygdala, caudate nucleus, hippocampus, and thalamus. No significant increases were observed in the nucleus accumbens, pallidum, and putamen. This finding is in partial accordance with previous reports of regenerated subcortical volumes in adolescent AN patients, except for the pallidum^20^. The lacking regeneration of the nucleus accumbens and the putamen in our sample might be explained by the difference in age between the samples and speaks to the hypothesis of a slower restoration in adult patients. The missing correlation between age and subcortical volumes seems to challenge this interpretation. However, lower scan-rescan reliability of these structures^49^ may obscure a potential influence. Long-term weight-recovered AN patients have been reported to show no differences in subcortical volumes^50^, suggesting that recovery of these regions may occur at a later stage.

Several biological mechanisms have been discussed as explanations for the observed gray matter reduction and subsequent regeneration in AN patients, such as dehydration^7,17,51^ or apoptosis (programmed cell death) of neurons or glia cells^52,53^. Recent reports on animal models of activity-based anorexia strongly support the hypothesis of glia cells playing a key role in AN^54,55^, suggesting that severe structural alterations in AN may be selectively linked to processes of astroglia and reduced glia cell proliferation^56^.

While the number of glia cells seems to stay relatively stable during healthy ageing^57^, the notion of diminished astrocyte proliferation^56^ offers an interesting explanation for the slower regeneration of cortical thickness in the older AN patients of the present study. Particularly the senescence of astrocytic cells, a protective response that permanently prevents cell proliferation^58,59^, increases with age^60^. The correlation between patients’ age and the reduced recovery of cortical thickness might thus be induced by ageing processes in glia cells, in particular diminished astrocyte proliferation. Younger AN patients would therefore have a greater capacity for full cortical restoration, which is in line with research on prognostic factors in AN, reporting better outcomes in younger patients^61^. In this respect, age may be a risk factor for persisting brain impairments and a chronic illness trajectory.

### Clinical implications

The findings of the present study provide strong evidence for weight restoration as an effective intervention to amend decreases of cortical and subcortical brain structures even in patients with severe AN. The strong correlation of age and regeneration of cortical thickness in the later treatment phase may indicate that younger patients have a better chance of complete cortical restitution. Age-dependent decreases in brain plasticity potentially aggravate the detrimental effects of malnutrition on the brain. This has two important clinical implications: (1) weight-restoration treatment should be started as early as possible to achieve better brain regeneration; and (2) a phase of continued guided stabilization of a healthy BMI (>18.5 kg/m^2^) after weight-restoration seems important to give patients time to sufficiently recover on both a psychological and a neural level. In fact, longer weight maintenance before discharge has previously been found to be predictive of a longer time period before rehospitalization^62^.

### Limitations

The strict weight criteria of the current study posed a challenge for patient recruitment and thus resulted in a moderate sample size. Nevertheless, our study sample is the largest to date investigating changes over three time points and the results are in line with previous structural studies with adult AN patients^3^. Furthermore, the longitudinal measurement of both AN patients and well-matched HC allowed us to account for potential normal age-related decreases of brain structures in HC^63–65^ and contributes to the reliability of the findings.

The age range of the current sample was 18−32 years. While this narrow age range offers the advantage of a homogeneous group, it limits generalizability. Further longitudinal studies, including a wider age range and a follow-up after weight stabilization, are needed to clarify the long-term influence of age in this context and to elucidate mediating factors of a successful recovery of AN patients.

### Conclusion

This systematic analysis of temporal patterns of structural brain recovery in adult AN patients suggests large-scale reversibility of reductions in cortical thickness and subcortical volumes, with the most pronounced changes occurring in the early stages of weight-restoration treatment. Patients’ age seems to have a notable influence on brain restitution, possibly reflecting decreases of brain plasticity as patients grow older.

## Supplementary information

Supplemental Information
