# Supplementary material for: Age influences structural brain restoration during weight gain therapy in anorexia nervosa
Source: Transl Psychiatry. 2020 May 4;10:126. doi: 10.1038/s41398-020-0809-7 (PMC7198513; doi:10.1038/s41398-020-0809-7)
Supplement: Supplementary file 1 — Supplemental Information [file 41398_2020_809_MOESM1_ESM.docx]

Supplemental Information

# Supplemental Methods

## Participants and study design

To calculate BMI, patients’ height was measured at the beginning of treatment. Weight measures were obtained twice per week before breakfast within the regular treatment scheme by trained hospital nurses. The range of BMI increases between TP1 and TP2 was 0.9-4kg/m^2^, and between TP2 and TP3 1-3.2kg/m^2^. Patients were assessed after a medical stabilization time of at least 2 weeks to mitigate the bias of under- or hyperhydration due to the condition of acute starvation of patients with AN ^1,2^. The mean duration between hospitalization and first scan was 4.88 ± 2.43 weeks. During this time, patients BMI increased on average by 0.91 ± 0.65kg/m^2^. Electrolyte levels for all patients were within the norm range (Sodium 136-14mmol/l; Potassium 3.3-4.5 mmol/l; Phosphate 0.87-1.45mmol/l; Chloride 98-107mmol/l; Calcium total 2.09-2.54mmol/l) and urine specific gravity indicated sufficient hydration (≤ 1.029) at TP1. Twelve patients were receiving medication when they entered the study: antidepressants (7), atypical antipsychotics and antidepressants (1), antidepressants and anxiolytics (1), atypical antipsychotics and antidepressants and/or anxiolytics (3). Patients receiving medication were instructed to continue taking them as prescribed (see Table S1 for further details).

Healthy controls (HC, BMI 18.5-23.0 kg/m^2^) were tested at two time points (TP1 and TP3) to ensure that potential time related changes could be disentangled from the pathological alterations due to anorexia nervosa (AN). To avoid effects of short-term hormone-dependent structural changes ^3^, both scans of HC were scheduled during the follicular phase of the women, within the first 10 days of their cycle. The weight of the HC was assessed at these two time points. Since higher BMI is associated with regional gray and white matter alterations ^4,5^, it was important for our study to select a group of healthy lean women as controls. All MRI scans were performed between 2 p.m. and 4.30 p.m.

Table S1

*Patients medication*

| Medications | TP1  (*n* = 24) | | TP2  (*n* = 26) | | TP3  (*n* = 26) | |
| --- | --- | --- | --- | --- | --- | --- |
|  | *n* | % | *n* | % | *n* | % |
| Atypical antipsychotics | 0 | 0 | 1 | 3.85 | 0 | 0 |
| Antidepressants | 7 | 29.17 | 5 | 19.23 | 8 | 30.77 |
| Atypical antipsychotics and antidepressants | 1 | 4.17 | 7 | 26.92 | 1 | 3.85 |
| Atypical antipsychotics and anxiolytics | 1 | 4.17 | 0 | 0 | 0 | 0 |
| Antidepressants and anxiolytics | 1 | 4.17 | 1 | 3.85 | 0 | 0 |
| Atypical antipsychotics, antidepressants, and anxiolytics | 2 | 8.33 | 1 | 3.85 | 2 | 7.69 |
| Total | 12 | 50.00 | 15 | 57.69 | 11 | 42.31 |
| *Note*. Antidepressants comprise selective reuptake inhibitors and tri-/tetracyclics. | | | | | | |

## MRI data acquisition

Whole-brain 3D T1-weighted structural images were acquired using a 3D Turbo-Field-Echo (TFE) sequence (echo time (TE) = 3.8 ms, repetition time (TR) = 8.3 ms, field of view (FOV) = 240 x 240 mm^2^, acquisition matrix = 240 x 240, 160 slices, isotropic voxel size = 1 mm^3^, flip angle = 8°, TFE factor = 240, duration = 4.5 min).

## Signal-to-noise ratio

The signal-to-noise ratio (SNR) in white matter of the longitudinal data as output from the FreeSurfer QA tools 1.1 (surfer.nmr.mgh.harvard.edu/fswiki/QATools) was compared between groups per time point (TP). The average SNR did not significantly differ between groups at TP1 (AN: mean = 24.13 ± 3.70, HC: mean = 23.80 ± 3.13; *t*(52) = 0.35, *p* = .73) or TP3 (AN: mean = 24.32 ± 3.55, HC: mean = 23.58 ± 2.98; *t*(54) = 0.84, *p* = .41)).

## Hedges’ g calculation

Hedges’ g was calculated with the means of the two groups (m1, m2), their respective standard deviations (sd1, sd2), and sample sizes (n1, n2) using the following formula ^6^:

$$\frac{m1 - m2}{\sqrt{\frac{(n1 - 1) * sd1^2 + (n2 - 1) * sd2^2}{n1 + n2 - 2}}}$$

This formula was used both for the between and the within group differences, as recommended by Dunlap et al. ^7^ to make effect sizes more comparable across studies.

# Supplemental Results

## Recovery of subcortical structures with weight restoration

Table S2

*Estimates of the linear mixed effect model of subcortical volume changes over the course of treatment*

|  | Group x Time | | | |
| --- | --- | --- | --- | --- |
| Region | df1 | df2 | F | *p^a^* |
| Gray matter (cortex) | 1 | 79.10 | 28.52 | <.00001 |
| Gray matter (subc.) | 1 | 78.22 | 34.36 | <.00001 |
| White matter | 1 | 78.44 | 2.73 | .307 |
| Cortico-spinal fluid | 1 | 78.34 | 34.90 | <.00001 |
| Intracranial volume | 1 | 84.94 | 0.001 | 1.0 |
| Accumbens nucleus | 1 | 79.60 | 0.22 | 1.0 |
| Amygdala | 1 | 78.35 | 14.41 | .002 |
| Caudate nucleus | 1 | 78.61 | 19.87 | <.001 |
| Hippocampus | 1 | 78.55 | 15.09 | .002 |
| Pallidum | 1 | 78.41 | 3.34 | .285 |
| Putamen | 1 | 78.37 | 4.63 | .172 |
| Thalamus | 1 | 78.51 | 41.21 | <.00001 |
| *Note*. Comparisons of subcortical volumes were performed with mean values controlled for total intra-cranial volume. ^a^ adjusted for multiple comparisons using Holm-Bonferroni correction ^8^. Subc. = subcortical. | | | | |

Table S3

*Subcortical brain* *volumes (corrected for total intra-cranial volume) per group and time point*

|  | Group and time point | | | | | | | | | |
| --- | --- | --- | --- | --- | --- | --- | --- | --- | --- | --- |
|  | AN1  (*n* = 24) | | AN2  (*n* = 26) | | AN3  (*n* = 26) | | HC1  (*n* = 30) | | HC3  (*n* = 30) | |
|  | *M* | *SD* | *M* | *SD* | *M* | *SD* | *M* | *SD* | *M* | *SD* |
| Accumbens nucleus | 0.26 | 0.05 | 0.26 | 0.05 | 0.26 | 0.05 | 0.30 | 0.07 | 0.30 | 0.06 |
| Amygdala | 1.02 | 0.11 | 1.04 | 0.11 | 1.04 | 0.11 | 1.08 | 0.13 | 1.08 | 0.13 |
| Caudate nucleus | 2.33 | 0.29 | 2.40 | 0.28 | 2.41 | 0.29 | 2.51 | 0.34 | 2.49 | 0.33 |
| Hippocampus | 2.67 | 0.25 | 2.72 | 0.24 | 2.75 | 0.25 | 2.91 | 0.27 | 2.92 | 0.27 |
| Pallidum | 1.28 | 0.12 | 1.27 | 0.13 | 1.27 | 0.11 | 1.33 | 0.15 | 1.35 | 0.15 |
| Putamen | 3.19 | 0.36 | 3.22 | 0.31 | 3.21 | 0.33 | 3.37 | 0.39 | 3.35 | 0.39 |
| Thalamus | 4.78 | 0.50 | 4.91 | 0.53 | 4.98 | 0.54 | 5.16 | 0.40 | 5.14 | 0.40 |
| *Notes*. Subcortical volumes were averaged across hemispheres. Mean values were corrected for total intra-cranial volume ((subcortical volume/intracranial volume) x 1000). | | | | | | | | | | |

## Correlations between brain restoration and clinical / demographic parameters

To explore potential influences on gray matter restoration, correlations between changes in cortical thickness or subcortical volumes and clinical parameters (BMI, BMI increase per week, duration of illness, eating disorder-related cognitions, and depression severity) as well as age at TP1 were computed within the group of AN patients (Table S4+S5).

Table S4

*Pearson correlations between changes in clinical parameters and restoration of subcortical volumes of anorexia nervosa patients*

| Measure | Accumbens | | Amygdala | | Caudate | | Hippocampus | | Pallidum | | Putamen | | Thalamus | |
| --- | --- | --- | --- | --- | --- | --- | --- | --- | --- | --- | --- | --- | --- | --- |
|  | *r* | *p* | *r* | *p* | *r* | *p* | *r* | *p* | *r* | *p* | *r* | *p* | *r* | *p* |
|  | TP2-TP1 | | | | | | | | | | | | | |
| BMI | .42 | .04 | .53 | .01 | .68 | <.01** | -.01 | .95 | -.10 | .64 | .17 | .42 | .57 | <.01 |
| BMI increase per week | .11 | .59 | .06 | .77 | .31 | .15 | -.34 | .10 | -.41 | .05 | .42 | .04 | .02 | .92 |
| Age | .21 | .32 | .16 | .46 | .20 | .35 | -.07 | .74 | -.25 | .24 | .40 | .05 | .07 | .75 |
| Duration of illness | -.04 | .86 | -.08 | .71 | -.13 | .53 | -.20 | .35 | -.15 | .49 | .09 | .66 | -.25 | .24 |
| EDE-Q total score | -.19 | .36 | -.30 | .15 | -.50 | .01 | .08 | .70 | -.03 | .88 | -.08 | .73 | -.32 | .13 |
| BDI score | -.07 | .74 | -.33 | .11 | -.41 | .05 | -.07 | .75 | -.07 | .74 | -.09 | .68 | -.22 | .30 |
|  | TP3-TP2 | | | | | | | | | | | | | |
| BMI | -.06 | .77 | -.01 | .97 | .30 | .14 | -.40 | .04 | .15 | .46 | .11 | .61 | -.26 | .21 |
| BMI increase per week | .38 | .06 | -.07 | .72 | .12 | .56 | .06 | .76 | -.12 | .56 | -.24 | .25 | -.06 | .79 |
| Age | .15 | .47 | -.34 | .09 | -.06 | .77 | -.06 | .75 | .25 | .23 | .01 | .96 | -.07 | .74 |
| Duration of illness | .20 | .32 | -.21 | .31 | .05 | .83 | .01 | .95 | .27 | .18 | .22 | .28 | -.10 | .64 |
| EDE-Q total score | .05 | .81 | -.32 | .13 | .19 | .38 | .44 | .03 | -.14 | .53 | .35 | .11 | .57 | <.01 |
| BDI score | -.02 | .94 | -.18 | .41 | .18 | .41 | .21 | .35 | -.04 | .85 | .34 | .12 | .35 | .10 |
| *Notes*. Correlations of subcortical structures were calculated with volumes averaged over both hemispheres, controlled for total intra-cranial volume. BMI = body mass index; BDI = Becks Depression Inventory total score; EDE-Q = Eating Disorder Examination Questionnaire score. °*p* < .10; **p* < .05; ***p* < .01; adjusted for multiple comparisons using Holm-Bonferroni correction ^8^. | | | | | | | | | | | | | | |

Table S5

*Pearson correlations between changes in clinical parameters and restoration of cortical thickness of anorexia nervosa patients*

| Measure | Cortical thickness L | | Cortical thickness R | | Cluster thickness L | | Cluster thickness R | |
| --- | --- | --- | --- | --- | --- | --- | --- | --- |
|  | *r* | *p* | *r* | *p* | *r* | *p* | *r* | *p* |
|  | TP2-TP1 | | | | | | | |
| BMI | .37 | .07 | .59 | <.01° | .42 | .04 | .59 | <.01* |
| BMI increase per week | .09 | .69 | .00 | .99 | .05 | .80 | -.02 | .92 |
| Age | -.19 | .39 | -.07 | .75 | -.21 | .33 | -.14 | .50 |
| Duration of illness | -.16 | .46 | -.13 | .55 | -.17 | .44 | -.16 | .44 |
| EDE-Q total score | -.25 | .23 | -.35 | .10 | -.28 | .18 | -.35 | .10 |
| BDI score | -.23 | .27 | -.32 | .13 | -.27 | .19 | -.32 | .12 |
|  | TP3-TP2 | | | | | | | |
| BMI | .35 | .08 | .28 | .16 | .32 | .12 | .24 | .23 |
| BMI increase per week | .36 | .07 | .46 | .02 | .46 | .02 | .42 | .03 |
| Age | -.59 | <.01* | -.59 | <.01* | -.58 | <.01* | -.53 | .01 |
| Duration of illness | -.38 | .06 | -.34 | .09 | -.38 | .05 | -.29 | .15 |
| EDE-Q total score | -.25 | .25 | -.23 | .29 | -.17 | .44 | -.14 | .53 |
| BDI score | -.12 | .60 | -.04 | .87 | -.11 | .62 | .06 | .79 |
| *Notes*. Cortical thickness: mean cortical thickness per hemisphere. Cluster thickness: mean cortical thickness within the clusters of significantly reduced cortical thickness in the group of AN patients, identified at TP1. BMI = body mass index; BDI = Becks Depression Inventory total score; EDE-Q = Eating Disorder Examination Questionnaire score; L = left; R = right; TP = time point. P-values represent the uncorrected values. P-values after adjustment for multiple comparisons using Holm-Bonferroni correction ^8^ are indexed with °*p* < .10; **p* < .05. | | | | | | | | |

To ensure that the association between differences in cortical thickness and age of patients with AN between TP2 and TP3 (Table S5) were not driven by general brain development, additional Pearson correlations were calculated for the group of HC. No statistically significant association was found (left hemisphere: r = -.15, p = .44, uncorrected; right hemisphere: r = -.17, p = .36, uncorrected). After removal of an extreme outlier (change of cortical thickness >3SD), these weak associations dropped to values close to zero (left hemisphere: r = -.01, p = .95, uncorrected; right hemisphere: r = -.001, p = .997, uncorrected), indicating that the influence of age on the regeneration of cortical thickness is present only in the AN group and not a result of general age effects.

Additional correlation analyses on patients current age demonstrated an association with illness duration (*r* =.72, *p* < .001) but not with illness severity, as indexed by patients BMI at admission (*r* =-.02, *p* = .92). With regard to the main findings, this indicates that while older patients had a longer illness duration, their illness was not systematically more severe in terms of weight loss.

## Influence of anorexia nervosa subtype

The sample consisted of 22 women with restrictive AN and 4 patients with the binge/purge subtype. Values of cortical thickness and subcortical gray matter did not differ systematically between the two subtypes (Fig. S1).

| 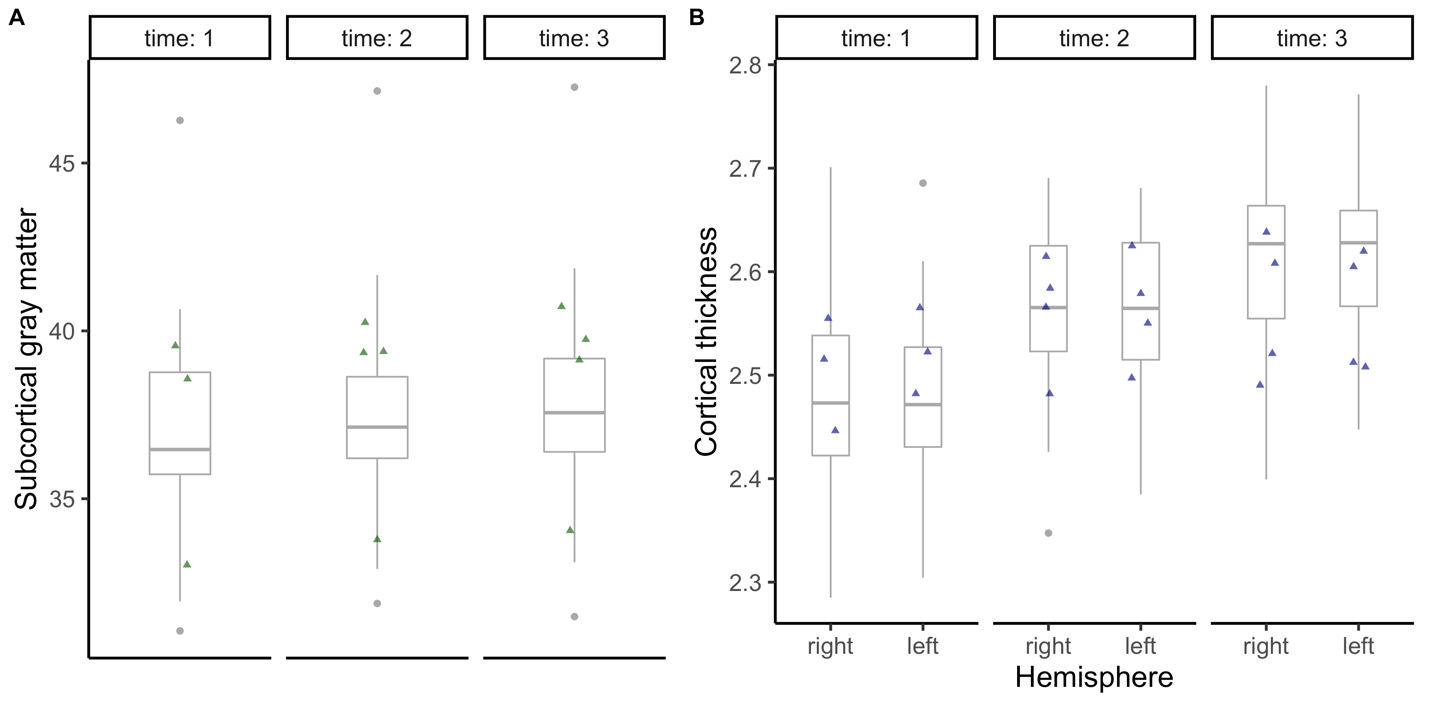 |
| --- |
| ***Figure S1***. Boxplots for AN patients with restrictive subtype, overlaid with values of patients with binge/purge subtype (triangles). The horizontal mark signifies the median per group, edges of the box represent 25^th^ and 75^th^ percentiles, and the whiskers extend to 1.5 interquartile ranges. ***A****:* Volume of subcortical gray matter [cm^3^] per time point. ***B****:* Global cortical thickness [mm] per time point and hemisphere. |

## Medication effects

Structural brain measures are susceptible to psychotropic medication, with atypical antipsychotics being associated with significant shrinkage of brain volume ^9,10^. To assess whether morphology of AN patients with medication differed from patients without medication with regard to cortical thickness and subcortical volumes, AN patients were divided into two subgroups (with and without current medication). T-test comparisons showed no differences in mean values of cortical thickness (*p*s ≥ .10) and subcortical volumes (*p*s ≥ .30) between the subgroups (Fig. S2), suggesting our findings of differences between AN patients and HC at TP1 and TP3 were not driven by current medication at the time of scanning. See Table S1 for details on medication type.

| 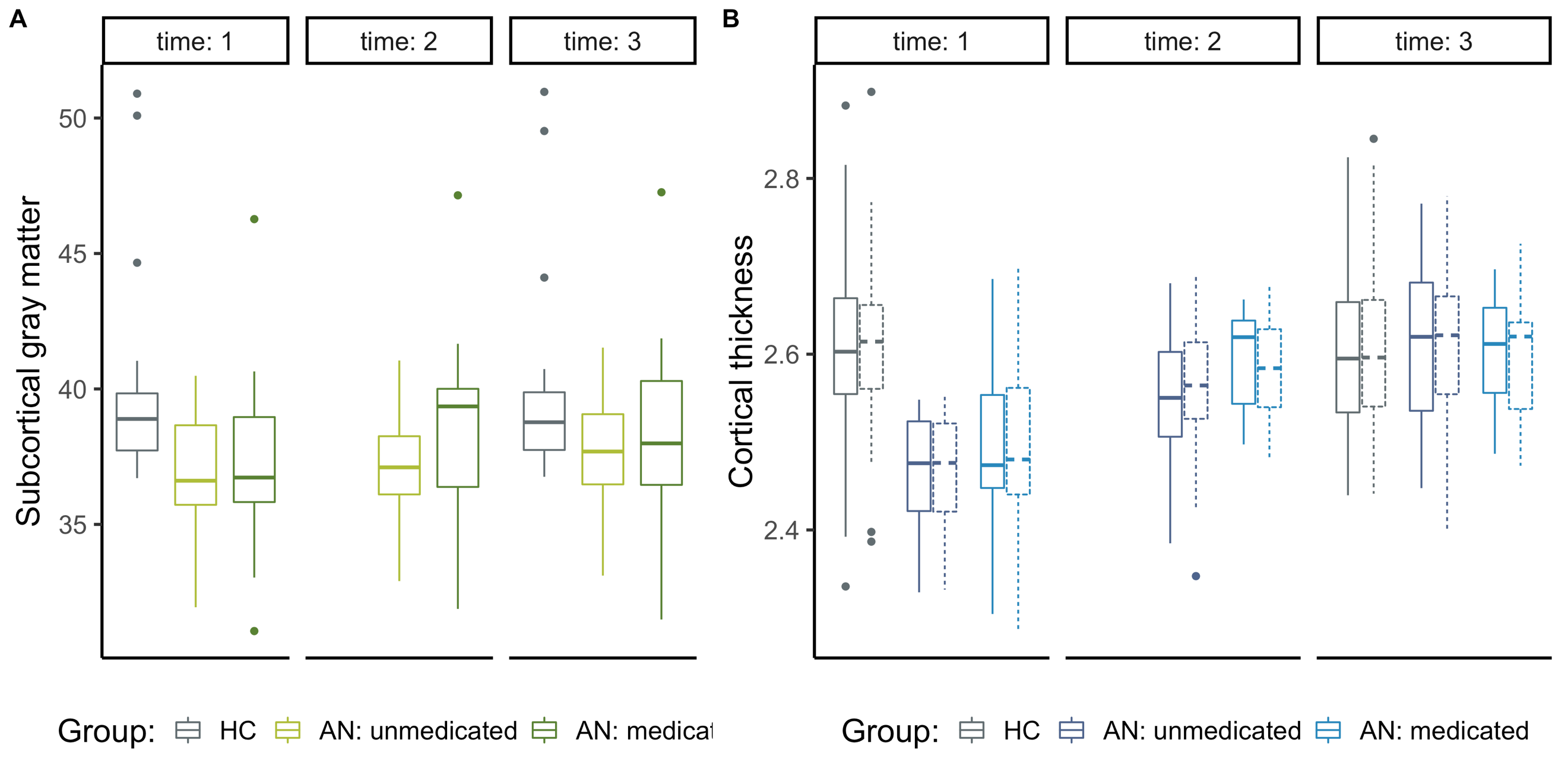 |
| --- |
| ***Figure S2***. Boxplots for HC and AN patients, with subgroups for medication use. The horizontal mark signifies the median per group, edges of the box represent 25^th^ and 75^th^ percentiles, and the whiskers extend to 1.5 interquartile ranges. ***A****:* Volume of subcortical gray matter [cm^3^] per group and time point. ***B****:* Global cortical thickness [mm] per group, time point, and hemisphere (solid line: left; dashed line: right hemisphere). |

## Association of age and cortical thickness at TP1

To investigate whether younger patients with AN showed a greater reduction of cortical thickness during the stage of acute starvation (TP1), Pearson correlations between patients’ age and cortical thickness at TP1 were calculated. No evidence for an association was found (left hemisphere, *r* = .06, *p* = .80, uncorrected; right hemisphere, *r* = -.04, *p* = .85, uncorrected). Similarly, the reduction of cortical thickness at TP1 of the younger half of patients, relative to their age-matched HCs (median difference HC-AN: left = 0.15 mm, right = 0.14 mm), barely differed from the older half of patients (median difference HC-AN: left = 0.12 mm, right = 0.13 mm; compare Fig. S3), suggesting that cortical thickness at the beginning of treatment was equally reduced in younger and older patients.

| 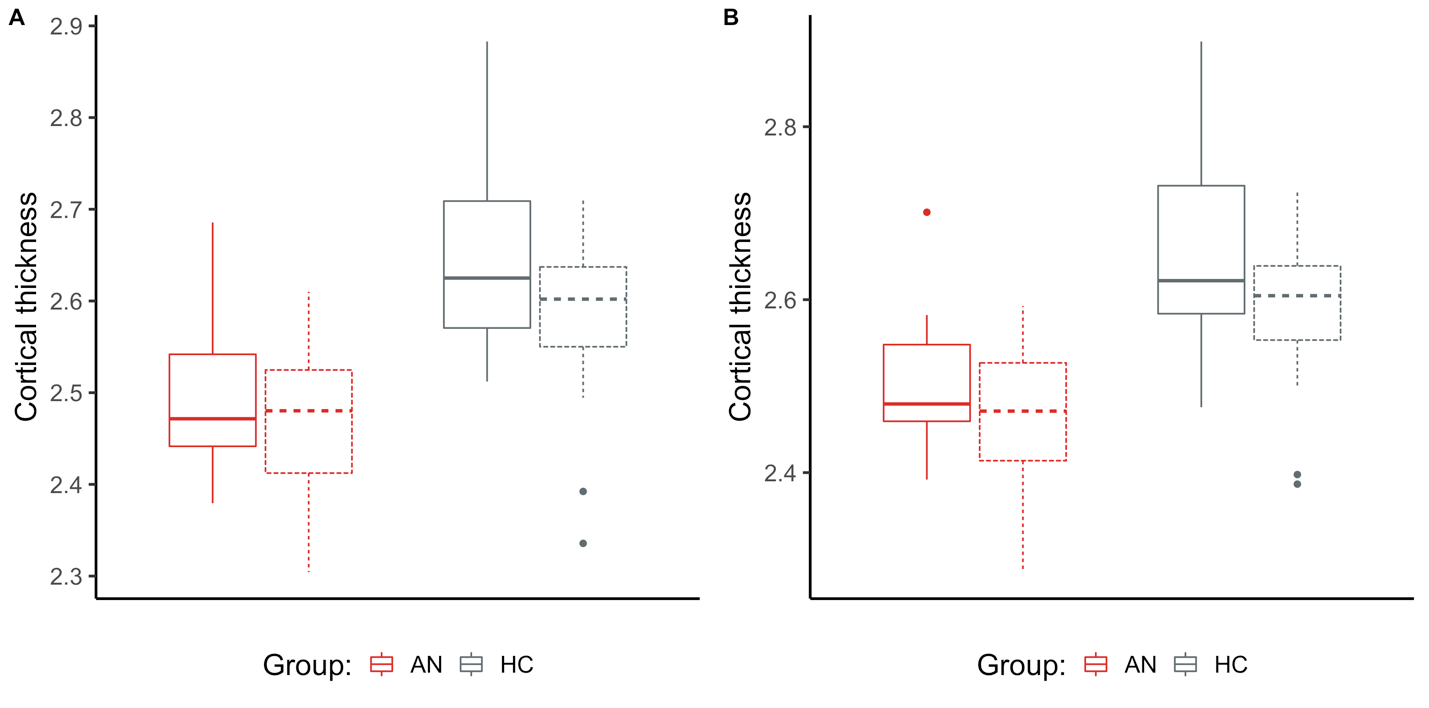 |
| --- |
| ***Figure S3***. Boxplots for cortical thickness [mm] per hemisphere (***A****:* left, ***B****:* right). Solid line: younger half of patients (aged 18-21) and age-matched healthy controls; dashed line: older half of patients (aged 22-32) and age-matched healthy controls. The horizontal mark signifies the median per group, edges of the box represent 25^th^ and 75^th^ percentiles, and the whiskers extend to 1.5 interquartile ranges. |

# References

1 King JA, Frank GKW, Thompson PM, Ehrlich S. Structural Neuroimaging of Anorexia Nervosa: Future Directions in the Quest for Mechanisms Underlying Dynamic Alterations. *Biol Psychiatry* 2018; **83**: 224–234.

2 Frank GKW, Favaro A, Marsh R, Ehrlich S, Lawson EA. Toward valid and reliable brain imaging results in eating disorders. *Int J Eat Disord* 2018; **51**: 250–261.

3 Comasco E, Sundström-Poromaa I. Neuroimaging the Menstrual Cycle and Premenstrual Dysphoric Disorder. *Curr Psychiatry Rep* 2015; **17**: 77.

4 Medic N *et al.* Increased body mass index is associated with specific regional alterations in brain structure. *Int J Obes* 2016; **40**: 1177–1182.

5 Medic N *et al.* BMI-related cortical morphometry changes are associated with altered white matter structure. *Int J Obes* 2019; **43**: 523–532.

6 Hedges L V. Distribution Theory for Glass’s Estimator of Effect Size and Related Estimators. *J Educ Stat* 1981; **6**: 107.

7 Dunlap WP, Cortina JM, Vaslow JB, Burke MJ. Meta-analysis of experiments with matched groups or repeated measures designs. *Psychol Methods* 1996; **1**: 170–177.

8 Holm S. A Simple Sequentially Rejective Multiple Test Procedure. *Scand J Stat* 1979; **6**: 65–70.

9 Dorph-Petersen K-A *et al.* The Influence of Chronic Exposure to Antipsychotic Medications on Brain Size before and after Tissue Fixation: A Comparison of Haloperidol and Olanzapine in Macaque Monkeys. *Neuropsychopharmacology* 2005; **30**: 1649–1661.

10 Lieberman JA. Antipsychotic Drug Effects on Brain Morphology in First-Episode Psychosis. *Arch Gen Psychiatry* 2005; **62**: 361.
